# Organ complications after CD19 CAR T-cell therapy for large B cell lymphoma: a retrospective study from the EBMT transplant complications and lymphoma working party

**DOI:** 10.3389/fimmu.2023.1252811

**Published:** 2023-09-27

**Authors:** Olaf Penack, Christophe Peczynski, Christian Koenecke, Emmanuelle Polge, Robin Sanderson, Ibrahim Yakoub-Agha, Nathalie Fegueux, Michael Daskalakis, Matthew Collin, Peter Dreger, Nicolaus Kröger, Urs Schanz, Adrian Bloor, Arnold Ganser, Caroline Besley, Gerald G. Wulf, Urban Novak, Ivan Moiseev, Hélène Schoemans, Grzegorz W. Basak, Christian Chabannon, Anna Sureda, Bertram Glass, Zinaida Peric

**Affiliations:** ^1^ Medical Clinic, Department for Haematology, Oncology and Tumorimmunology, Charité Universitätsmedizin Berlin, Berlin, Germany; ^2^ EBMT Transplant Complications Working Party, Paris, France; ^3^ EBMT Paris Study Office, Department of Haematology, Saint Antoine Hospital, Paris, France; ^4^ INSERM UMR-S 938, Sorbonne University, Paris, France; ^5^ Department of Hematology, Hemostasis, Oncology and Stem Cell Transplantation, Hannover Medical School, Hannover, Germany; ^6^ Kings College Hospital, Departement of Haematological Medicine, London, United Kingdom; ^7^ CHU de Lille, Univ Lille, INSERM U1286, Infinite, Lille, France; ^8^ CHU Lapeyronie, Département d`Hématologie Clinique, Montpellier, France; ^9^ Department of Hematology, University Hospital Bern, Bern, Switzerland; ^10^ Department of Oncology, University Hospital Bern, Bern, Switzerland; ^11^ Adult HSCT Unit, Northern Centre for Bone Marrow Transplantation, Newcastle upon Tyne, United Kingdom; ^12^ Department of Hematology, University of Heidelberg, Heidelberg, Germany; ^13^ Bone Marrow Transplantation Centre, University Hospital Eppendorf, Hamburg, Germany; ^14^ Clinic of Hematology, University Hospital, Zurich, Switzerland; ^15^ Christie NHS Trust Hospital, Adult Leukaemia and Bone Marrow Transplant Unit, Manchester, United Kingdom; ^16^ Department of Haematology, Hemostasis, Oncology, Hannover Medical School, Hannover, Germany; ^17^ Department of Paediatric Oncology, Bristol Royal Hospital for Children, Bristol, United Kingdom; ^18^ Department of BMT, Bristol Royal Hospital for Children, Bristol, United Kingdom; ^19^ Universitaetsmedizin Goettingen, Klinik für Hämatologie und Medizinische Onkologie, Göttingen, Germany; ^20^ Department of Medical Oncology, Inselspital, Bern University Hospital, University of Bern, Bern, Switzerland; ^21^ Department of Hematology, First Pavlov State Medical University of St. Petersburg, St. Petersburg, Russia; ^22^ Department of Hematology, University Hospitals Leuven and KU Leuven, Leuven, Belgium; ^23^ Department of Hematology, Oncology and Internal Medicine, The Medical University of Warsaw, Warsaw, Poland; ^24^ EBMT Cellular Therapy and Immunobiology Working Party, Leiden, Netherlands; ^25^ Institut Paoli-Calmettes Comprehensive Cancer Centre, Inserm CBT-1409, Aix-Marseille Université, Marseille, France; ^26^ Clinical Hematology Department, Institut Català d’Oncologia-Hospitalet, Barcelona, Spain; ^27^ Institut de Ciències Biomèdiques de Bellvitge (IDIBELL), Universitat de Barcelona, Barcelona, Spain; ^28^ EBMT Lymphoma Working Party, Leiden, Netherlands; ^29^ Department of Hematology, Oncology, and Tumor ImmunologyKlinikum Berlin-Buch, Helios, Berlin, Germany; ^30^ University Hospital Centre Zagreb and School of Medicine, University of Zagreb, Zagreb, Croatia

**Keywords:** organ complications, CAR T-cell, large B-cell lymphoma, CD19, toxicity

## Abstract

We investigated ≥ grade 3 (CTC-AE) organ toxicities for commercial CD19 chimeric antigen receptor T cell (CAR-T cell) products in 492 patients (Axi-Cel; n = 315; Tisa-Cel; n = 177) with Large B-cell Lymphoma in the European Society for Blood and Marrow Transplantation (EBMT) CAR-T registry. The incidence of ≥ grade 3 organ toxicities during the first 100 days after CAR-T was low and the most frequent were: renal (3.0%), cardiac (2.3%), gastro-intestinal (2.3%) and hepatic (1.8%). The majority occurred within three weeks after CAR-T cell therapy. Overall survival was 83.1% [79.8-86.5; 95% CI] at 3 months and 53.5% [49-58.4; 95% CI] at one year after CAR-T. The most frequent cause of death was tumour progression (85.1%). Non-relapse mortality was 3.1% [2.3-4.1; 95% CI] at 3 months and 5.2% [4.1-6.5; 95% CI] at one year after CAR-T. The most frequent causes of non-relapse mortality were cell-therapy-related toxicities including organ toxicities (6.4% of total deaths) and infections (4.4% of total deaths). Our data demonstrates good safety in the European real-world setting.

## Background

CAR-T cells that target CD19 have become standard treatments of relapsed or refractory large B cell lymphoma (rrLBCL) in 3^rd^ line or beyond, and have moved up to 2^nd^ line following the recent reports of two positive randomized trials (1, 2). The safety of CAR-T cells is of major concern, since this new class of anti-tumour therapy may have unknown side effects. The complete risk profile of CD19+ CAR T-cells has not been fully defined by the relatively small registration trials leading to approval of CAR T-cell products. Therefore, health authorities obliged Marketing Authorization Holders (MAH) to report efficacy and toxicity data on predefined numbers of patients treated with commercial CART products in the real world-setting. The EBMT registry that was established forty years ago in Europe to collect data on patients receiving autologous or allogeneic hematopoietic cell transplants, has recently extended its capacity to the capture of long-term follow-up (up to 15 years) of patients treated with CAR-T Cells through the design and implementation of a Cellular Therapy Form (CTF). Primary use of data allows for the conduct of studies such as the one reported in this manuscript, while secondary use of data allows for the conduct of post-authorization safety studies (PASS) and post-authorization efficacy studies (PAES) in partnership with MAH/Pharmas or other stakeholders. Classical side effects of CD19 CAR-T cell products, which are already well described in clinical cohorts, include cytokine release syndrome (CRS), immune cell associated neurotoxicity syndrome (ICANS) and B-cell aplasia. Currently, there is emerging evidence of additional both short-term and medium-term unwanted effects, which were in part unknown at the time of regulatory approval (3–6). There is evidence on neurological events other than ICANS, including ischemic attacks, peripheral neuropathy and Alzheimer’s dementia (7). The patho-mechanism behind this association is not known. In one study major cardiovascular events occurred in 17% of patients till one month after CAR-T cell infusion (8) and in another study frequent adverse events were observed within one year after CAR-T cell infusion (4). The most frequent pulmonary toxicity symptom observed in previous studies was hypoxia, but also pleural effusion, pulmonary embolism, allergic rhinitis and pneumomediastinum were described (4). In a recent study by Wudikarn and colleagues metabolic toxicities after CART have been detected as a frequent complication (4). Typical electrolyte abnormalities were hypokalemia, hypophosphatemia and hypocalcemia. Hyperglycemia and hypoglycemia were also frequent (4, 9, 10).

In the current study, we have used the EBMT CAR-T cell registry (11) to assess the real-world outcome of CD19 CAR-T cell products. Our study aimed at investigating (non-CRS and non-neurotoxicity) organ complications of CD19 CAR-T cell products in adult patients with LBCL reported to the EBMT.

## Material and methods

### Study design and data collection

This is a retrospective multicentre analysis using the dataset of the EBMT registry. The EBMT is a not-for-profit professional association uniting more than 600 transplant centres that are required to register and report regular follow up on all consecutive hematopoietic cell transplantations, and since the recent and first approvals in the summer of 2018 of patients treated with approved CAR-T Cells. In the CAR-T cell registry of the EBMT, a growing fraction of commercial CAR-T cell therapies in Europe are registered, and data on outcome is provided. Audits are routinely performed to determine the accuracy of the data. The study was planned and approved by the Transplant Complications Working Party of the EBMT and by the EBMT board. All patients gave their written informed consent to collect, transfer and use their medical information for research purposes. The study was conducted in accordance with the Declaration of Helsinki and Good Clinical Practice guidelines.

Eligibility criteria for this analysis included patients 18 years of age or older undergoing CD19+ CAR-T cell therapy for LBCL before the end of July 2021. Only patients with an available status on organ toxicities during the first 100 days after CAR-T were included. Data on organ toxicities were collected via a form designed for the post-authorization studies on CAR-T cell therapy. In this form, occurrence, time of onset and grading of renal, cardiac, hepatic, gastro-intestinal, skin, pulmonary toxicities, as well as hypogammaglobulinemia are reported. For the current analysis, we have exclusively included the occurrence of grades 3-4 severe toxicities based on CTCAE criteria (12). The full CTCAE criteria are publicly available for download: https://ctep.cancer.gov/protocoldevelopment/electronic_applications/docs/ctcae_v5_quick_reference_5x7.pdf.

### CAR-T cell products

Patients were treated with commercial products and had received axicabtagene ciloleucel (Axicel, 64%) or tisagenlecleucel (Tisacel, 36%). Both are autologous anti-CD19 T cell products containing a second-generation CAR. Axicel is generated by transduction of apheresed and immunoselected blood CD3+ lympohocytes with a retroviral vector and contains a CD28 co-stimulatory domain. Tisacel is manufactured with a lentiviral vector, and contains a CD137 (4-1BB) costimulatory domain.

### Statistical methods

The frequency of severe grade 3-4 organ complications according CTCAE occurring in the first 100 days was given for the following organs: kidney, heart, liver, gut, skin and lungs. The time from CAR-T cell infusion to the occurrence of each complication, as well as the grade of the complication, were also described.

Additional study endpoints were Overall Survival (OS), Progression-Free Survival (PFS), Relapse Incidence (RI) and Non-Relapse Mortality (NRM). Start time was the date of CAR-T cell infusion for all endpoints. NRM was defined as death without relapse/progression, PFS was defined as survival without relapse or progression. Probability of OS and PFS were calculated using the Kaplan-Meier method. Cumulative incidence functions were used to estimate NRM and Relapse Incidence in a competing risk setting, death and relapse competing with each other (13).

Statistical analyses were performed with R 4.1.2 software (R Development Core Team, Vienna, Austria) packages.

## Results

### CAR-T, disease, and patient characteristics

We analysed adult patients with LBCL receiving CD19+ CAR-T cell therapy before the end of July 2021 who were reported to the EBMT and had information on organ toxicities. We identified 492 patients fulfilling the inclusion criteria. Population characteristics are shown in **Table 1**. Since the first CART cells were approved in the summer of 2018, most patients in this study received CAR-T cell infusion in 2019 (42%) or 2020 (45%). The majority of patients were from the United Kingdom, France, Germany or Switzerland. Median age was 61.6 years (range 18.7-81) and 61% were male. Karnofsky performance score was 90 or higher in 61% of the patients for whom the information was available (363/492).

**Table 1:** Baseline characteristics of the cohort.

Baseline parameters	Absolute Nr. (%)
Country	
United Kingdom	139 (28)
France	114 (23)
Germany	98 (20)
Switzerland	79 (16)
Spain	31 (6)
Italy	14 (3)
Czech Republic	10 (2)
Israel	4 (1)
Greece	3 (1)
Median Age (min-max) [IQR]	61.6 (18.7-81) [52.8-69.3]
Male	300 (61)
Female	192 (39)
Karnofsky Score >= 90	222 (61)
Karnofsky Score < 90 Missing	141 (39) 129
No previous transplant	366 (74)
Previous auto transplant only	111 (23)
Previous allo transplant only	9 (2)
Previous allo and auto transplant	6 (1)
Lymphodepletion chemotherapy	
Fludarabine/cyclophosphamide	481 (98
Other	10 (2)
Missing	1
Disease Status before CART	
Chemorefractory/Progressive disease (PD)	369 (75)
Untreated relapse	34 (7)
Stable disease (no change, no response)	22 (5)
Partial remission	45 (9)
Complete remission (CR) Missing	21 (4) 1
Median follow-up (months) [95% CI]	12.5 [12.2-13]
CAR T-cell product	
Yescarta™ (axicel)	315 (64)
Kymriah™ (tisacel)	177 (36)
Number of prior lines of treatment	
1	43 (10)
2	126 (28)
3	166 (37)
4	60 (13)
5 or more	47 (10)
missing	50

Most patients received CAR-T cells without having a previous transplantation (74%). Disease status before CAR-T cell therapy was mainly chemorefractory/progressive disease (75%). Most patients had received either two (28%) or three (37%) previous lines of therapy. However, 23% had four or more lines of previous therapy.

**Table 2** describes pre-existing comorbidities in the cohort. 48.7% of patients had at least one pre-existing comorbidity present before CAR-T infusion. The most frequently recorded comorbidity was pulmonary (19.3%), followed by diabetes (7.5%), cardiac (7.3%), obesity (5.9%), solid tumour (5.6%) and psychiatric disturbance (4.7%).

**Table 2:** Baseline co-morbidities of the cohort.

Baseline parameters		Absolute Nr. (%)
**Comorbidies conditions present at or before CAR-T**	No	238 (51.3%)
	Yes	226 (48.7%)
	missing	28
**Solid tumour, previously present**	No	401 (94.4%)
	Yes	24 (5.6%)
	missing	67
**Inflammatory Bowel Disease, previously present (IBD)**	No	418 (97.4%)
	Yes	11 (2.6%)
	missing	63
**Rheumatologic comorbidity**	No	416 (98.1%)
	Yes	8 (1.9%)
	missing	68
**Infections present before CAR-T**	No	410 (96.2%)
	Yes	16 (3.8%)
	missing	66
**Diabetes (requiring treatment other than diet alone)**	No	395 (92.5%)
	Yes	32 (7.5%)
	missing	65
**Renal comorbidity (moderate to severe)**	No	406 (97.4%)
	Yes	11 (2.6%)
	missing	75
**Hepatic comorbidity**	No	411 (97.9%)
	Mild	9 (2.1%)
	missing	72
**Arrythmia (conduction blocks)**	No	395 (93.6%)
	Yes	27 (6.4%)
	missing	70
**Cardiac comorbidity**	No	393 (92.7%)
	Yes	31 (7.3%)
	missing	68
**Cerebrovascular disease: Stroke/CNS haemorrhage**	No	416 (99.5%)
	Yes	2 (0.5%)
	missing	74
**Heart valve disease**	No	405 (96.4%)
	Yes	15 (3.6%)
	missing	72
**Pulmonary comorbidity**	No	338 (79.7%)
	Yes (grade unknown)	8 (1.9%)
	Mild	8 (1.9%)
	Moderate	40 (9.4%)
	Severe	30 (7.1%)
	missing	68
**Obesity**	No	446 (94.1%)
	Yes	28 (5.9%)
	missing	18
**Peptic ulcer**	No	418 (99.5%)
	Yes	2 (0.5%)
	missing	72
**Psychiatric disturbance**	No	402 (95.3%)
	Yes	20 (4.7%)
	missing	70

### Occurrence of organ toxicities until day +100 after CD19+ CART infusion

We were able to collect data on time of onset as well as severity of renal, cardiac, hepatic, gastro-intestinal, skin and pulmonary toxicities. The occurrences of grades 3-4 (CTC-AE) severe toxicities in the different organs are given in **Table 3**.

**Table 3:** Occurrence of severe organ toxicities until day +100 and patients alive at day +100 after CD19+ CART infusion.

	Organ Toxicity present Number (%)	Patients still alive at day +100 Number (%)	No Organ Toxicity Number (%)	Patients still alive at day +100 Number (%)	Missing data Number of patients
Renal	14 (3)	6 (42.9)	459 (97)	375 (79.3)	19
Cardiac	11 (2.3)	8 (72.7)	461 (97.7)	373 (79)	20
Gastro-intestinal	11 (2.3)	7 (63.6)	464 (98.7)	373 (79.2)	17
Hepatic	8 (1.8)	4 (50)	462 (98.2)	374 (79.6)	22
Skin	3 (0.6)	3 (100)	463 (99.4)	373 (80)	26
Pulmonary	2 (0.5)	1 (50)	467 (99.5)	376 (80.2)	23

Overall, the frequency of severe organ toxicities was relatively low. Severe renal toxicity occurred most frequently (3.0%) followed by severe cardiac, gastro-intestinal and hepatic toxicities (2.3%, 2.3% and 1.8% respectively). Skin toxicity and pulmonary toxicity were only observed in three and two patients, respectively. Data in **Table 2** suggest that patients with these severe complications might have a lower chance of survival at day +100 after CAR-T cell therapy as compared to patients without organ complications. We can indeed observe that among the 14 patients developing renal toxicity during the first 100 days, only six were still alive at day 100. Similarly, eight patients were reported as having had liver toxicity during the first 100 days and only four of them were still alive at day 100. However, the numbers of patients with severe organ toxicities were too low to perform advanced statistics. **Table 4** shows the proportion of patients with organ toxicities receiving either axi-cel (Yescarta) or tisa-cel (Kymriah) as well as the proportion according to age, using a 60-year cut off.

**Table 4:** Organ toxicities according to administered product and to patient age.

	Organ Toxicity n=492 cases Number (%)	Yescarta n=315	Kymriah n=177	<= 60 years n=223	> 60 years n=269
Renal	14 (3)	7	7	6	8
Cardiac	11 (2.3)	9	2	3	8
Gastro-intestinal	11 (2.3)	5	6	5	6
Hepatic	8 (1.8)	2	6	3	5
Skin	3 (0.6)	1	2	1	2
Pulmonary	2 (0.5)	1	1	0	2

223 patients were <= 60 years old and 269 patients were > 60 years old.

**Figure 1:**
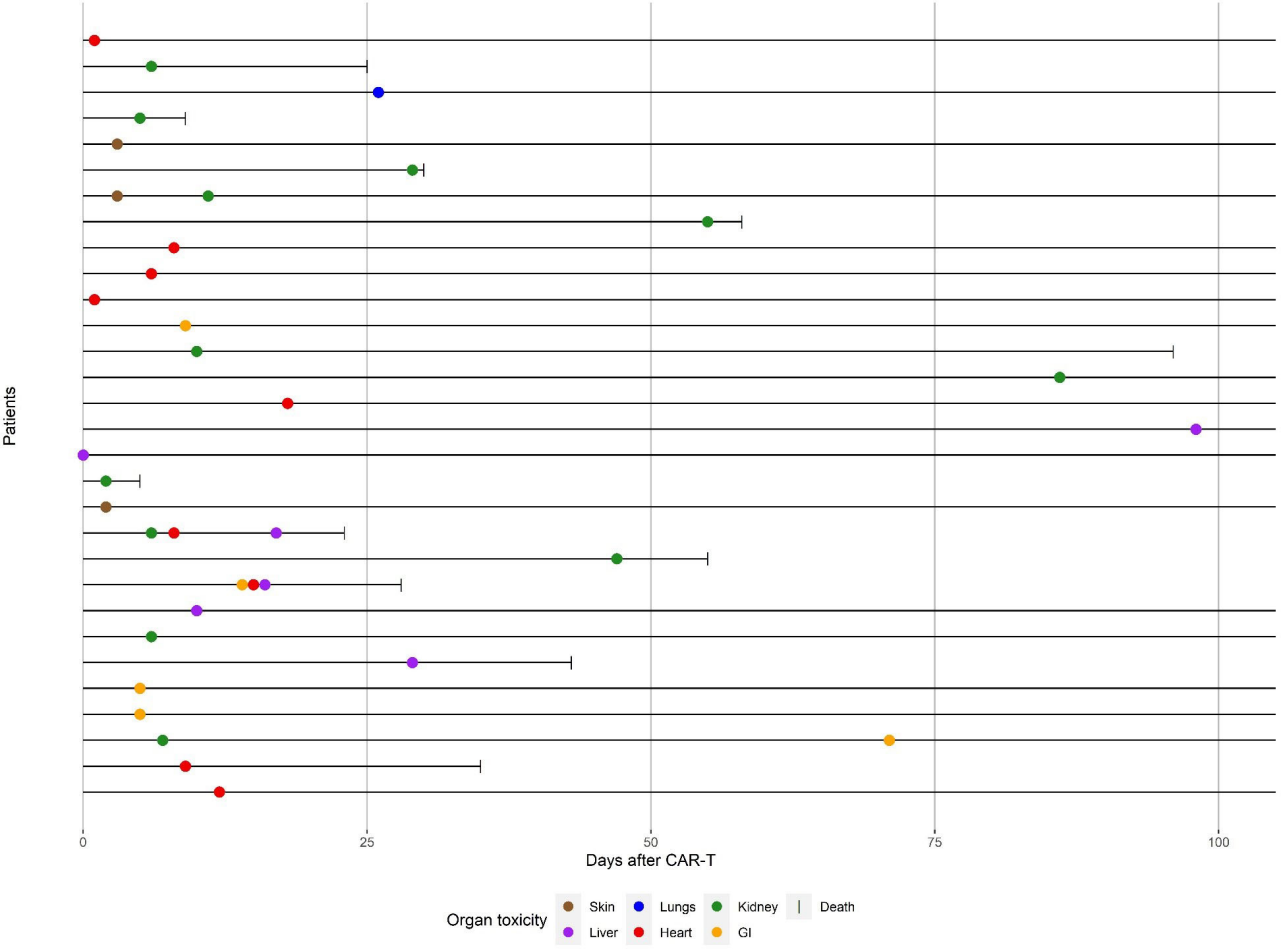
Timing of severe organ toxicities and deaths until day +100 after CD19+ CART infusion.

The occurrence of organ complications in relation to time of occurrence and death events in individual patients is visualized in **Figure 1**. The majority of severe organ complications occurred before day+20, during the early phase after CAR-T cell therapy. However, some renal, hepatic and gastro-intestinal complications occurred at a later time point after CAR-T cell infusion, between day+30 and day+100. The cumulative incidences of cytokine release syndrome (CRS) were the following:

- At 3 days [95% CI] 46.6% [41.8-51.3]
- At 6 days [95% CI] 81.5% [77.5-84.9]
- At 9 days [95% CI] 93% [90.1-95]
- At 12 days [95% CI] 96.5% [94.2-97.9]

### Occurrence of hypogammaglobulinemia until day +100 after CD19+ CART infusion

Hypogammaglubulinemia, (<4g/l) which is paradigmatic of an on target/off tumour effect for CAR-T cells targeting CD19, was observed in 236 patients (57.3%; data missing for 80 cases). Of note, most of those patients (n = 170, 80.2%) had already low IgG levels before CAR-T cell therapy. 87/178 (48.9%, data missing for 58 cases) of the patients have seen their hypogammaglobulinemia worsened following CAR-T cell infusion. Hypogammaglobulinemia resolved in the first 100 days in only 21 (10.7%) patients (data was missing for 39 patients).

### Mortality and reasons of death after CD19+ CAR-T cell therapy

In the whole population, we found that NRM was 3.1% [2.3-4.1; 95% CI] at 3 months and 5.2% [4.1-6.5; 95% CI] at one year after CAR-T cell therapy. Overall survival was 83.1% [79.8-86.5; 95% CI] at 3 months and 53.5% [49-58.4; 95% CI] at one year. Progression-free survival was 64.9% [62.5-67.4; 95% CI] at 3 months and 36.5% [33.9-39.2; 95% CI] at one year. Finally, relapse incidence was 32% [29.6-34.4; 95% CI] at 3 months and 58.3% [55.6-61; 95% CI] at one year after CAR-T cell therapy.

A description of the causes of death is given in **Table 5**. The most frequent cause of death by far was relapse of LBCL (85.1%). The most frequent causes of NRM were toxicities in close connection to the infused CAR-T cell product (cell-therapy-related toxicities including CRS, ICANS, and organ toxicity; 6.4% of total deaths) and infections (4.4% of total deaths). Organ complications, that were likely to be not cell-therapy related (0.4% of total deaths), as well as secondary malignancies (0.4% of total deaths) occurred less frequently. Of note, the decision if NRM was due to organ toxicity related *vs*. unrelated to the CAR-T cell infusion was done by the centre and no clear definition existed.

**Table 5:** Main causes of death.

Original Disease with or without complications	212	(85.1%)
Cell Therapy Related	16	(6.4%)
Other organ toxicity in absence of relapse	1	(0.4%)
Infections in absence of relapse	11	(4.4%)
Secondary malignancy	1	(0.4%)
Other	8	(3.2%)
Missing	4	

## Discussion

Our present study is the first analysis of EBMT real-world data on other organ complications than CRS and ICANS after anti-CD19 CAR-T cell therapy for LBCL. Our main finding is that despite high rates of CRS in this dataset a low frequency of severe organ complications occurred, marginally contributing to a relatively low NRM. Of note, disease status before CAR-T cell therapy was mainly chemorefractory/progressive disease (75%) in the patient population reported here, which is considerably higher than in the registration trials. Despite this difference, our present results are mostly in line with follow up data of the initial CAR-T cell trials in patients with LBCL, also showing low NRM and manageable safety (9, 14). However, some of the more frequent organ toxicities found in our present study, such as renal, cardiac and hepatic complications, were not reported in the pivotal Zuma-1 (Axi-Cel) and Juliet (Tisa-Cel) trials. This is most likely due to larger patient numbers and a longer follow up period in our real-world analysis. As expected, our patients in this retrospective analysis had received more prior lines of treatment and had a lower performance status when compared to the patient populations in the two registration trials conducted to support marketing approval of Tisa-Cel and Axi-Cel. These different patient characteristics may also be another relevant aspect explaining the occurrence of organ toxicities in real-world conditions.

Recently, a real world data set of patients with LBCL undergoing CAR-T cell therapy in Germany has been published (n=356) (5). When comparing NRM in this national analysis with our current data, differences in NRM become evident. At a median follow up of 11 months, NRM was 10% in the German analysis *vs*. 4.2% NRM with 12 months median follow up in our current analysis. Interestingly, the reasons for death were also different with bone marrow aplasia and infections as the driving factor for NRM in the recent German analysis (5) and organ toxicities being the major factor for NRM in our current analysis. We recently found in LBCL patients receiving CD19+ CAR-T cells, that severe cytopenia had no significant impact on overall survival (HR 1.13 [95%CI 0.74-1.73] p=0.57) (15). However, patients with severe cytopenia had a poorer progression-free survival (HR 1.54 [95%CI 1.07-2.22] p=0.02) and a higher relapse incidence (HR 1.52 [95%CI 1.04-2.23] p=0.03). Again, the most likely reason for the pronounced differences in outcome between the two analyses is the difference in patient characteristics. The German analyses reported that only 13% of patients in their dataset fulfilled the inclusion criteria of the Zuma-1 trial, indicating a population at very high risk for toxicities and for NRM. Organ complications were not reported in detail in this publication, making it impossible to compare them to our present study. Also recently, a larger real world data set from France was published mainly comparing results from Axi-Cel *vs*. Tisa-Cel treated patients, but again organ complications were not reported in detail so far (16). In line, reporting of organ complications was not done in detail so far for the recent trials comparing CAR-T cell therapy to standard of care as second line option for large B-cell lymphoma (17, 18).

There are publications from centres in the USA with detailed information on organ toxicities. The MD Anderson Cancer Center in Houston looked specifically at cardiac complications in a retrospective analysis of 165 patients with LBCL undergoing therapy with axicel or tisacel (19). Sixteen percent developed at least one major adverse cardiac event until day +30. Among them, three myocardial infarctions. However, the occurrence of cardiac events was not associated to NRM. The Memorial Sloan-Kettering Cancer Center published even more comprehensive data on organ complications in a smaller series of patients (n=60) undergoing CD19+ CAR-T cell therapy for LBCL (4). At one year follow up, they found severe cardiovascular, pulmonary, hepatic and gastro-intestinal toxicities in 20%, 13%, 13% and 10% of patients respectively. Again, NRM was low with 1.7% at one year and was not associated to the occurrence of organ complications. **Table 6** summarizes the occurrence of organ toxicities in major trials on CD19+ CAR-T cells. Although it is impossible to directly compare the previous trial results to our current data it appears that the previously reported complication rate was relatively high. One reason could be the improved clinical management in the more recent period, but we are unable to rule out underreporting of complications in the EBMT data base (see below).

**Table 6:** Organ complications (other than CRS and ICANS) reported in major trials on CD19+ CAR-T cells.

Reference *Year*	Product	Disease	Patients (n)	Neurologic	Pulmonary	Cardiologic	Metabolic	Prolonged cytopenia	Infection	Immune-related	Secondary Tumor	Other
Phase II ELIANA 2018 (20)	Tisa-Cel	ALL	75 trial		Hypoxia 24%		Hypokalemia 27% Hypophosphatemia 24%	37% day 28 37%	Febrile neutropenia 35% till week 8			Tumor lysis syndrome 4% Aspartate- aminotransferase increased 27% Bilirubin increased 17%
Cordeiro et al. (7)	Tisa-Cel Axi-Cel	ALL NHL CLL	86 trial	10%				16% after day+90	61%	8%	29% of patients in continuous CR	Hypogammaglobulinemia (IgG <400 mg/dL or i.v immunoglobulin m (IVIG) replacement, observed in 67%
Zuma-1 and -9 (21)	Axi-Cel	LBCL	31 trial	Fatigue 53% Headache 46% Confused state 27% Dizziness 21% Somnolece 17%	Hypoxia 31% Cough 29% Dyspnea 21% Pleural effusion 16%	Hypotension 58% Tachycardia 40% Peripheral edema 19% Tachycardia 19% Hyper-tension 16%	Hypocalcemia 40% Hyponataemia 35% Hypokalemia 33% Hypophosphatemia 29% Hyperglycemia 19% Hypomagnesemia 19%	48% by day +30 11% at 2 years	28% grade II or worse			
Juliet (10)	Tisa-Cel	LBCL	93 trial	Dizziness 13% Anxiety 12% Fatigue 28%	Dyspnea 19% Cough 19%	Hypotension 29% Tachycardia 12% Peripheral edema 17%	Hypokalemia 23% Hypomagnesemia 19%, Hypophosphatemia 19%	D+28 32%	D+28 20% infections			

NHL, Non-Hodgins Lymphoma, CLL – Chronic Lymphocytic Leukemia.

Blank spaces where left for parameters not reported in the respective publications.

A limitation of our study is that we were not able to perform advanced statistical analyses on the association of severe organ toxicities and NRM because of the low frequencies of these two events, despite the fact that this is one of the largest patient population ever studied. A possible further limitation of our current results is the retrospective data acquisition. We were therefore unable to monitor primary data to exclude a possible underreporting of organ toxicities in the EBMT data file. Finally, the follow up period was limited and is too short to adequately describe the occurrence of later toxicities – e.g. secondary malignancies.

A different and relevant way of looking at our current results is to compare them to toxicity and NRM data in allogeneic stem cell transplantation, which remains a relevant alternative treatment option in patients with relapsed/refractory LBCL. In a recent analysis, we found 12.2% NRM one year after allogeneic stem cell transplantation (22). Although the patient populations can’t be directly compared, the much lower 5.2% NRM one year after CD19+ CAR-T cell therapy in our current study suggests differences in safety in favour of CAR-T cells.

In conclusion, we found a low frequency of severe organ complications and a relatively low NRM after CAR-T cell therapy for LBCL in this international, multicentre analysis. These data demonstrate good safety in the European real-world setting.

## Data availability statement

The raw data supporting the conclusions of this article will be made available by the authors, without undue reservation.

## Ethics statement

The studies involving humans were approved by Transplant Complications Working Party of the EBMT, EBMT board. The studies were conducted in accordance with the local legislation and institutional requirements. Written informed consent for participation was not required from the participants or the participants’ legal guardians/next of kin in accordance with the national legislation and institutional requirements.

## Author contributions

All authors listed have made a substantial, direct, and intellectual contribution to the work and approved it for publication.

## References

[1] AbramsonJSSolomonSRArnasonJJohnstonPBGlassBBachanovaV. Lisocabtagene maraleucel as second-line therapy for large B-cell lymphoma: primary analysis of the phase 3 TRANSFORM study. Blood (2023) 141:1675–84. doi: 10.1182/blood.2022018730 36542826

[2] Westin JR Oluwole OO Kersten MJ Miklos DB Perales MA Ghobadi A. Survival with axicabtagene ciloleucel in large B-cell lymphoma. N Engl J Med (2023) 389:148–57. doi: 10.1056/NEJMoa2301665 37272527

[3] Penack O Koenecke C. Complications after CD19+ CAR T-cell therapy. Cancers (Basel) 12 (2020). doi: 10.3390/cancers12113445 PMC7699604 33228221

[4] Wudhikarn K Pennisi M Garcia-Recio M Flynn JR Afuye A Silverberg ML. DLBCL patients treated with CD19 CAR T cells experience a high burden of organ toxicities but low nonrelapse mortality. Blood Adv (2020) 4:3024–33. doi: 10.1182/bloodadvances.2020001972 PMC7362382 32614964

[5] Bethge WA Martus P Schmitt M Holtick U Subklewe M von Tresckow B. GLA/DRST real-world outcome analysis of CAR-T cell therapies for large B-cell lymphoma in Germany. Blood (2022). doi: 10.1182/blood.2021015209 35316325

[6] Korell F Penack O Mattie M Schreck N Benner A Krzykalla J. EASIX and severe endothelial complications after CD19-directed CAR-T cell therapy-A cohort study. Front Immunol (2022) 13:877477. doi: 10.3389/fimmu.2022.877477 35464403 PMC9033201

[7] Cordeiro A Bezerra ED Hirayama AV Hill JA Wu QV Voutsinas J. Late events after treatment with CD19-targeted chimeric antigen receptor modified T cells. Biol Blood Marrow Transplant (2020) 26:26–33. doi: 10.1016/j.bbmt.2019.08.003 31419568 PMC6953906

[8] Lefebvre B Kang Y Smith AM Frey NV Carver JR Scherrer-Crosbie M. Cardiovascular effects of CAR T cell therapy: A retrospective study. JACC CardioOncol (2020) 2:193–203. doi: 10.1016/j.jaccao.2020.04.012 32776016 PMC7413146

[9] Locke FL Ghobadi A Jacobson CA Miklos DB Lekakis LJ Oluwole OO. Long-term safety and activity of axicabtagene ciloleucel in refractory large B-cell lymphoma (ZUMA-1): a single-arm, multicentre, phase 1-2 trial. Lancet Oncol (2019) 20:31–42. doi: 10.1016/S1470-2045(18)30864-7 30518502 PMC6733402

[10] Schuster SJ Bishop MR Tam CS Waller EK Borchmann P McGuirk JP. Tisagenlecleucel in adult relapsed or refractory diffuse large B-cell lymphoma. N Engl J Med (2019) 380:45–56. doi: 10.1056/NEJMoa1804980 30501490

[11] McGrath E Chabannon C Terwel S Bonini C Kuball J. Opportunities and challenges associated with the evaluation of chimeric antigen receptor T cells in real-life. Curr Opin Oncol (2020) 32:427–33. doi: 10.1097/CCO.0000000000000665 32665456

[12] U.D.o. Health and H. Services, common terminology criteria for adverse events (CTCAE) version 5.0 (2017). Available at: https://ctep.cancer.gov/protocolDevelopment/electronic_applications/ctc.htm.

[13] Fine JP Gray RJ. A proportianal hazards model for the subdistribution of a competing risk. J Am Stat Assoc (1999) 94:496–509. doi: 10.1080/01621459.1999.10474144

[14] Schuster SJ Tam CS Borchmann P Worel N McGuirk JP Holte H. Long-term clinical outcomes of tisagenlecleucel in patients with relapsed or refractory aggressive B-cell lymphomas (JULIET): a multicentre, open-label, single-arm, phase 2 study. Lancet Oncol (2021) 22:1403–15. doi: 10.1016/S1470-2045(21)00375-2 34516954

[15] Penack O Peczynski C Koenecke C Polge E Kuhnl A Fegueux N. Severe cytopenia after CD19 CAR T-cell therapy: a retrospective study from the EBMT Transplant Complications Working Party. J Immunother Cancer (2023) 11. doi: 10.1136/jitc-2022-006406 PMC10124318 37072350

[16] Bachy E Le Gouill S Di Blasi R Sesques P Manson G Cartron G. A real-world comparison of tisagenlecleucel and axicabtagene ciloleucel CAR T cells in relapsed or refractory diffuse large B cell lymphoma. Nat Med (2022) 28:2145–54. doi: 10.1038/s41591-022-01969-y PMC9556323 36138152

[17] Kamdar M Solomon SR Arnason J Johnston PB Glass B Bachanova V. Lisocabtagene maraleucel versus standard of care with salvage chemotherapy followed by autologous stem cell transplantation as second-line treatment in patients with relapsed or refractory large B-cell lymphoma (TRANSFORM): results from an interim analysis of an open-label, randomised, phase 3 trial. Lancet (2022) 399:2294–308. doi: 10.1016/S0140-6736(22)00662-6 35717989

[18] Locke FL Miklos DB Jacobson CA Perales MA Kersten MJ Oluwole OO. Axicabtagene ciloleucel as second-line therapy for large B-cell lymphoma. N Engl J Med (2022) 386:640–54. doi: 10.1056/NEJMoa2116133 34891224

[19] Steiner RE Banchs J Koutroumpakis E Becnel M Gutierrez C Strati P. Cardiovascular events in patients treated with chimeric antigen receptor t-cell therapy for aggressive B-cell lymphoma. Haematologica (2021). doi: 10.3324/haematol.2021.280009 PMC9244830 34758610

[20] Maude SL Laetsch TW Buechner J Rives S Boyer M Bittencourt H. Tisagenlecleucel in children and young adults with B-cell lymphoblastic leukemia. N Engl J Med (2018) 378:439–48. doi: 10.1056/NEJMoa1709866 PMC5996391 29385370

[21] Strati P Varma A Adkins S Nastoupil LJ Westin J Hagemeister FB. Hematopoietic recovery and immune reconstitution after axicabtagene ciloleucel in patients with large B-cell lymphoma. Haematologica (2020). doi: 10.3324/haematol.2020.254045 PMC8485681 32732355

[22] Penack O Peczynski C Mohty M Yakoub-Agha I Styczynski J Montoto S. How much has allogeneic stem cell transplant-related mortality improved since the 1980s? A retrospective Anal EBMT. Blood Adv (2020) 4:6283–90. doi: 10.1182/bloodadvances.2020003418 PMC7756984 33351121

