# Supplementary material for: Organ complications after CD19 CAR T-cell therapy for large B cell lymphoma: a retrospective study from the EBMT transplant complications and lymphoma working party
Source: Front Immunol. 2023 Sep 27;14:1252811. doi: 10.3389/fimmu.2023.1252811 (PMC10565347; doi:10.3389/fimmu.2023.1252811)
Supplement: Supplementary file 1 [file DataSheet_1.docx]

Supplementary table 1: Main causes of death.

| Original Disease with or without complications | 212 | (85.1%) |
| --- | --- | --- |
| Cell Therapy Related | 16 | (6.4%) |
| Other organ toxicity in absence of relapse | 1 | (0.4%) |
| Infections in absence of relapse | 11 | (4.4%) |
| Secondary malignancy | 1 | (0.4%) |
| Other | 8 | (3.2%) |
| Missing | 4 |  |
